# Pseudouridine and *N*1-methylpseudouridine as potent nucleotide analogues for RNA therapy and vaccine development

**DOI:** 10.1039/d4cb00022f

**Published:** 2024-03-19

**Authors:** Lyana L. Y. Ho, Gabriel H. A. Schiess, Pâmella Miranda, Gerald Weber, Kira Astakhova

**Affiliations:** a Technical University of Denmark 2800 Kongens Lyngby Denmark kiraas@kemi.dtu.dk; b The Hong Kong Polytechnic University 11 Yuk Choi Rd Hung Hom Hong Kong; c Departamento de Física, Universidade Federal de Minas Gerais Belo Horizonte MG Brazil; d Programa Interunidades de Pós-Graduação em Bioinformática, Universidade Federal de Minas Gerais Belo Horizonte MG Brazil

## Abstract

Modified nucleosides are integral to modern drug development, serving as crucial building blocks for creating safer, more potent, and more precisely targeted therapeutic interventions. Nucleobase modifications often confer antiviral and anti-cancer activity as monomers. When incorporated into nucleic acid oligomers, they increase stability against degradation by enzymes, enhancing the drugs’ lifespan within the body. Moreover, modification strategies can mitigate potential toxic effects and reduce immunogenicity, making drugs safer and better tolerated. Particularly, *N*1-methylpseudouridine modification improved the efficacy of the mRNA coding for spike protein of COVID-19. This became a crucial step for developing COVID-19 vaccine applied during the 2020 pandemic. This makes *N*1-methylpseudouridine, and its “parent” analogue pseudouridine, potent nucleotide analogues for future RNA therapy and vaccine development. This review focuses on the structure and properties of pseudouridine and *N*1-methylpseudouridine. RNA has a greater structural versatility, different conformation, and chemical reactivity than DNA. Watson–Crick pairing is not strictly followed by RNA that has more unusual base pairs and base-triplets. This requires detailed structural studies and structure–activity relationship analyses for RNA, also when modifications are incorporated. Recent successes in this direction are revised in this review. We describe recent successes with using pseudouridine and *N*1-methylpseudouridine in mRNA drug candidates. We also highlight remaining challenges that need to be solved to develop new mRNA vaccines and therapies.

## Abstract 2

*N*1-Methylpseudouridine, and its “parent” analogue pseudouridine, are potent nucleotide analogues for RNA therapy and vaccine development.

## Graphical abstract

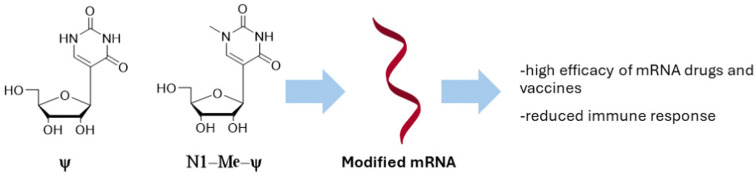

## Introduction

Deoxyribo- and ribonucleic acids (DNA and RNA) represent a class of natural compounds central to the life on Earth. Their main functions are to store, transmit genetic information and to allow species adopt, survive, and evolve.

**Fig. 1:**
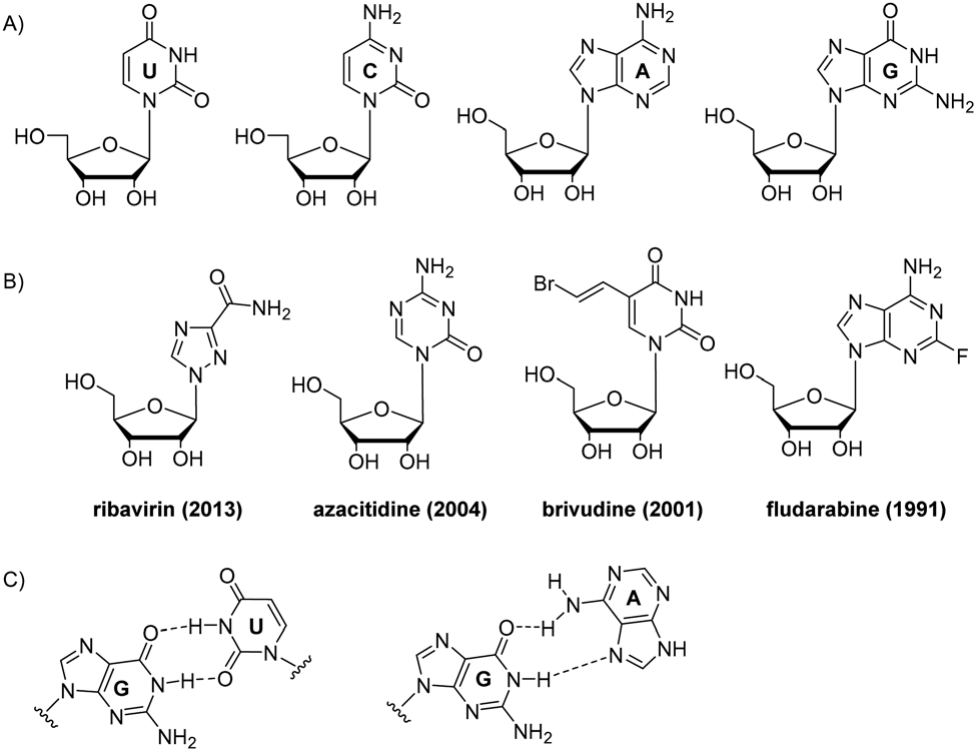
Chemical structures of natural RNA nucleotides (A), examples of nucleoside drugs (year of FDA approval) (B), and wobble RNA base pairs: GU and GA (C).

Nucleosides are monomers of DNA and RNA (the latter illustrated in Fig. 1A). They have two distinct structural elements: nitrogen heterocycle, called nucleobase, and a ribose carbohydrate. Each element serves as a scaffold for multiple non-covalent interactions, vital to biological performance of nucleic acids. Nucleobase provides with π-stacking and hydrogen bonding interactions, forming nucleic acid helices and universal genetic code.

Given crucial biological importance of nucleic acids, interference with their synthesis and function creates powerful therapeutic interventions. This can be achieved *via* chemical modification of nucleosides.

Multiple nucleoside drugs with nucleobase modifications have been developed. Several examples are shown in Fig. 1B. Modified nucleoside monomers include drugs for treating viral infections (ribavirin, idoxuridine, trifluridine, brivudine), cancer (azacitidine, fludarabine, 6-mercaptopurine, decitabine), and immunosuppressive drug azathioprine, used in organ transplantation and autoimmune diseases.^1^ Due to their altered nucleobase structure, these drugs interfere with DNA synthesis, transcription and/or translation leading to cell death.^1^

As to oligonucleotide drugs, it mostly ribose and phosphate backbone modifications that have been incorporated. Chemical modifications in these drugs result in enhanced stability, improved pharmacokinetics, reduced toxicity, increased target specificity, or resistance to degradation, thereby improving their therapeutic potential.^2^

Over the last two decades, there has been a growing interest in RNA drug development. RNA differs from DNA by having a 2′-hydroxyl group in ribose sugar, which gives rise to unique conformational features, specific hydration, and electrostatic properties of RNA (Fig. 1A).^1^ Another structural feature of RNA is that uracil nucleobase does not contain the major groove 5-methyl group as that in DNA nucleotide thymidine. As a result of this, RNA duplexes are more compact than B-DNA helices. Ribose nucleotides tend to adopt the C3′-*endo* sugar pucker resulting in compact A-form RNA duplexes with eleven base pairs per turn.^1^

RNA has various alternative base pairings, such as G:U wobble pair and A:C pair, which differ from DNA. G:U pair has an exocyclic amino group that acts as a prominent hydrogen bond donor in the minor grooves, important for recognising proteins and other ligands (Fig. 1C).^3^ G:U pair frequently occurs, such as in codon–anticodon interaction which forms the genetic code between tRNA and mRNA. The hydrogen bonding of G:U are strongly dependent on their next neighbour pairs, ranging from very weak bonding when in tandem configuration to strong if located at the end of a double strand.^4^ G:A pairs are ubiquitous at RNA helices termini, in loops and folds of tertiary structure. Among G:A, G:G and A:A pairs, sheared G:A pairs that include a Hoogsteen interaction are most common (Fig. 1C).^3^

RNA nucleotides form multiple interactions with nucleobases and sugars. G-tetrad and G-quartet are similar for DNA and RNA but more diversity is found in RNA. Quadruple interaction can be formed in highly folded RNA molecules as well.^3^

RNA folding is complex and sensitive to temperature and buffer composition. Metal ions like Mg^2+^ and K^+^ stabilize RNA tertiary structure.^3^ As a result of stable structure, misfolding of RNA could become a severe problem with *e.g.*, therapeutics and vaccine candidates.^3^

Pseudouridine (Ψ) is an abundant post-transcriptional modification of mRNA which together with *N*1-methylpseudouridine (*N*1-Me-Ψ), became a milestone in developing mRNA drug candidates. This review describes Ψ and *N*1-Me-Ψ nucleobase analogues, their impact on RNA structure and appealing properties in RNA vaccines and therapeutic candidates. We also give highlights on most potent applications of modified mRNA achieved so far, discuss future directions and remaining challenges.

### Pseudouridine

**Fig. 2:**
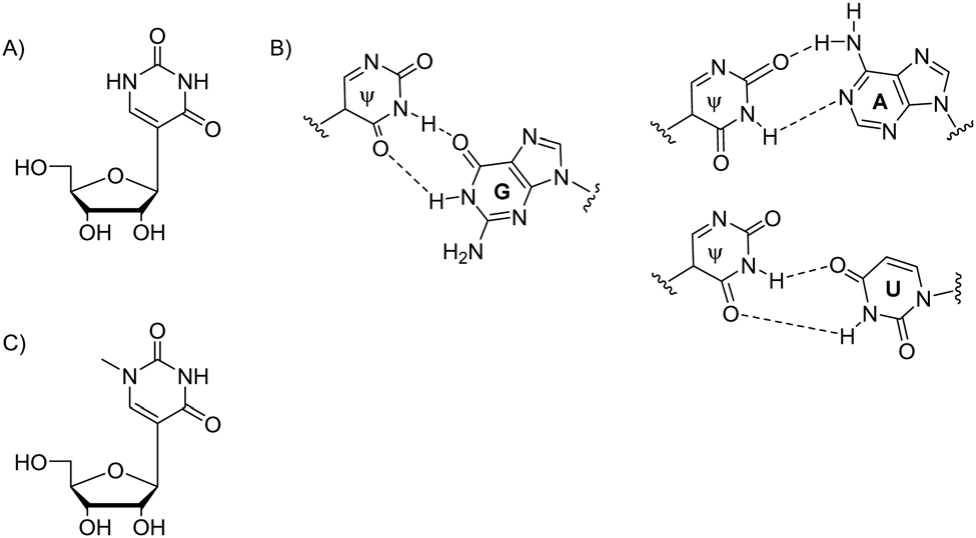
Chemical structure of Ψ (A), its base pairing with G, A and U nucleobases (B); chemical structure of *N*1-methyl-Ψ (C).

The first known and one of the most abundant RNA modifications is the pseudouridine (Ψ), or 5-ribosyluracil, which is a post-transcriptional modification and an isomer of uridine (U) (Fig. 2A). Pseudouridine contains a C–C base-sugar bond due to the uracil base is attached to the sugar by a C1′–C5 bond unlike a C1′–N1 glycosidic linkage (Fig. 2), enhancing the base rotation. Moreover, it has an additional ring nitrogen atom (N1 imino atom), which behaves as an additional hydrogen bond donor.^5,6^ The replacement of U by Ψ promotes a C3′-*endo* sugar conformation and increases the local base stacking, thermodynamically stabilizing RNA duplexes.^5,7,8^

Pseudouridine (Ψ) sites have been identified in different types of natural mRNA, such as tRNA, rRNA, snRNA and mRNA.^9–13^ Intracellular Ψ formation is catalyzed by a class of enzymes known as pseudouridine synthases.^14^ Pseudouridine synthases can be classified into two main families: stand-alone pseudouridine synthases and pseudouridine synthase domains within larger proteins. Stand-alone pseudouridine synthases include TruA found in bacteria and TruB found in bacteria and yeast. In eukaryotes, several pseudouridine synthase domains have been found. Eucaryotic H/ACA box small nucleolar ribonucleoproteins (snoRNPs) have a dyskerin (Cbf5) component which catalyzes pseudouridylation in rRNA, snRNA, and telomerase RNA. Nop10 is another component of H/ACA snoRNPs which is involved in the pseudouridylation activity.^14^

In humans, there are multiple enzymes catalyzing pseudouridylation. RPUSD (RNA pseudouridylate synthase domain-containing) family consists of several enzymes involved in Ψ formation. RPUSD1 and RPUSD2 are involved in mitochondrial RNA pseudouridylation. The PUS family consists of enzymes with pseudouridine synthase activity towards multiple RNA species, represented by PUS1 and PUS7.

Quantitative Ψ detection remains being challenging. Therefore its biological role is still not completely understood. Incorporated into mRNA, Ψ increases its stability and modifies various cellular and biological processes, such as transcription, pre-mRNA splicing and mRNA translation.^14^ Ψ was also found in stop codons.^15^

Pseudouridylation is a crucial post-transcriptional modification that influences the structural stability and function of transfer RNA (tRNA) as well. With nanopore sequencing it has been shown that Ψ modifications are present in diverse positions of tRNA.^16^ As a result of a modification in the hisT gene, the tRNA *E. coli* and *Salmonella typhimurium* lacks Ψ38, Ψ39 and Ψ40 positions and may have a reduced polypeptide chain elongation rate (20–25%) and longer cell division time (30%). In addition, inhibition of Ψ38 and Ψ39, as a result of DEG1 gene disruption, in *S. cerevisiae* leads to reduced growth rate.^9^

Notably, pseudouridylation of tRNA affects its methylation at other positions. In tRNA^Phe^ of *S. cerevisiae*, Ψ55 positively influences the introduction of methylated nucleotides, m^5^U54 and m^1^A58.^16^

Aberrant pseudouridine modifications in tRNA can impact translation accuracy and efficiency, potentially contributing to disease pathogenesis.^10,11,17–20^ For example, mutations in the PUS1 gene, which encodes a pseudouridine synthase, are associated with mitochondrial myopathy and sideroblastic anaemia.^19^ Abnormal pseudouridylation has been implicated in neurological disorders.^20^ Altered tRNA modifications, including pseudouridylation, have been observed in various cancer types.^9,10,17,18^

Nucleic acid function is guided by non-covalent interactions between polynucleotides and with cellular protein machinery.^3^ Therefore 2D and 3D structure of RNA is central to its interactions and function. Regarding impact on RNA structure, two hydrogen bond donors by N1 and N3 imine protons, and two hydrogen bond acceptors are present in Ψ (Fig. 2B). Therefore, Ψ acts as a universal nucleobase by enhancing stability when it pairs with A, G, U or C in a double helix. The relationship between Ψ-modified mRNA structure and stability is supported by multiple examples of *in vitro*-synthesised, more stable, Ψ-containing mRNAs, and the natural Ψ-containing mRNAs’ increased half-life time, as in eukaryotic parasite *Toxoplasma gondii*.^21,22^

NMR structural studies found that Ψ stacks better than U due to the C1′–C5 glycoside bond in Ψ that has higher rotational lability than the C1′–N1 bond in U.^5,23^ Ψ enhances local RNA base stacking in both single- and double-stranded conformations and promotes an increase in neighbouring stacking stabilization at nucleotide level.^7,9^ In addition, Ψ induces a more rigid phosphodiester backbone in its vicinity, increasing the base stacking neighbouring, perhaps due to C–C isomerization in Ψ.^9,14^ Moreover, the base stacking enhancement is considered the most important contribution of Ψ to RNA stability.^7,24–27^

To investigate more the impact of Ψ on RNA structure, thermodynamic studies were conducted.^5^ The data revealed that Ψ stabilizes the RNA duplexes when U is replaced and Ψ–A, Ψ–G, Ψ–U and Ψ–C pairs are formed. The effect of pseudouridylation is more significant when it occurs at the internal positions of the RNA structure, usually releasing more negative free energy. Enhanced thermodynamic stability by Ψ is around 0.5 kcal mol^−1^, except in 5′-CΨG/3′GAC and 5′-GΨC/3′CGG, nearly 2.5 and 1.5 kcal mol^−1^ is recorded respectively. Ψ contribution is, in most cases, weaker than a typical hydrogen bond (2–10 kcal mol^−1^), which indicates that the enhanced stability is a result of a different mechanism.^28^ This endorses the hypothesis that the contribution of Ψ to the stability arises from the C1′–C5 bond properties. Current NMR data^5^ suggest that the N1H in the 5′-CΨG/3′-GAC trimer is in a weak hydrogen bond while there is no evidence for hydrogen bonding in the 5′-GΨC/3′-CGG trimer.^5^ Ψ biological roles, such as nonsense suppression of stop codons and non-synonymous translation, might be better understood by identifying contexts where N1H is in an active hydrogen bond.^29^ The 3D structures and computational studies of these outlier duplexes could be interesting to investigate due to little knowledge of the interactions that are favourable to stacking.

Ψ–A, Ψ–G, Ψ–U and Ψ–C base pairs have shown enhancement of base stacking and also sequence context dependence.^5,6,30^ High level quantum mechanical (QM) methods have shown a clear dependence of the change in the base stacking energies concerning the sequence context with a range from −1.59 to 0.23 kcal mol^−1^. However, the change from U–A to Ψ–A base pair not always stabilizes the stacking interactions of the duplex.^6,30^ Yet experimental data of internal and terminal Ψ–A pair has shown greater stability in comparison to predicted data of U–A pair in the same duplexes, on average 1.7 and 1.0 kcal mol^−1^, respectively.^30^

Terminal Ψ–A, –C, –G and –U pairs show stability enhancement in both 5′ and 3′ terminals in a similar way to U–A, –C, –G and –U pairs, respectively. Curiously, those Ψ pairs at 3′ terminal reach slightly higher energies than the U pairs, except Ψ–A pair. Ψ–C pair contributes with more stability than U–C pair only when added at 3′ terminal. On the other hand, only Ψ–U pair shows a more stable behaviour than U pair in 5′ terminal. Similarly, Ψ–G pairs have higher stabilization when at 3′ terminal (3.22 kcal mol^−1^) in comparison to U–G pairs (2.44 kcal mol^−1^). Finally, any internal Ψ pair enhances the thermodynamic stability of RNA duplexes. Mainly Ψ–A and Ψ–G pairs have large energies, within a 0.4–0.8 kcal mol^−1^, when compared to A–U and G–U pairs, perhaps due to the favourable base stacking and hydrogen bonds of Ψ.^5,31^

### RNA *N*1-methylpseudouridine structure

*N*1-Methylpseudouridine (*N*1-methyl-Ψ) is another naturally occurring RNA nucleotide analogue^32^ which has an extra hydrogen bond donor in the nucleobase (Fig. 2C). This *N*1-modified structure can be found in natural 18S rRNA and tRNA, not only in humans but also in archaea and eukaryotes. Research shows that RNAs of Thermococcales and Nanoarchaea^33^ include *N*1-methyl-Ψ.^34,35^*N*1-Methyl-Ψ biosynthetic pathway begins with converting U to Ψ, being catalyzed by the aforementioned pseudouridine synthase Pus10.^36,37^*S*-Adenosylmethionine (SAM)-dependent pseudouridine *N*3-methyltransferase YbeA in eubacteria^38,39^ and *N*1-specific pseudouridine methyltransferase Nep1 are two enzymes that can further methylate Ψ. Nep1 catalyse the *N*1-specific pseudouridine methylation of position 1191 (*Saccharomyces cerevisiae* numbering, nucleotide 913 in *M. jannaschii*) in RNA.^40,41^

Ψ- and *N*1-methyl-Ψ modified RNA duplexes have different stability which has been confirmed with multiple thermal denaturation studies.^5^*N*1-Methyl-Ψ differs from Ψ by having a methyl group to replace the extra hydrogen at *N*1-position, so it no longer has the universal base character like Ψ (Fig. 2). *N*1-Methyl-Ψ only forms the traditional Watson–Crick pair but both Ψ and *N*1-methyl-Ψ have the C5–C1′ bond that allows rotation between the nucleobase and sugar, achieving better base-pairing, base-stacking and duplex stability.^42^

A recent molecular dynamics study shows that *N*1-methyl-Ψ induces a higher stabilization effect of the dsRNA, due to stronger stacking and base pair interactions, than Ψ. Moreover, *N*1-methyl-Ψ:A pair have a stronger binding interaction than both U:A and Ψ:A pairs in the majority of neighbours context.^43^

Overall, *N*1-methyl-Ψ is more like uridine in translation coding but at the same time, behaves like Ψ that let the mRNA not trigger the immune response which is of critical importance to RNA vaccines and therapies, described below.

### mRNA drug development

mRNA holds a potential to treat a vast spectrum of diseases and to act as a vaccine, by producing the desired protein *in vivo* and acting as adjuvant.^44^ Key steps in mRNA drug development include: sequence design, chemical modification, formulation, testing *in vitro* and *in vivo*, and finally, trials.

Currently there are two approved mRNA vaccines, by Moderna and Pfizer.^45,46^ According to FDA, January 2024, there are 56 mRNA drugs in the clinical pipeline worldwide, with R&D mainly focused on vaccines, accounting for about 84%, while therapeutic drugs account for about 16%. Except for the mRNA COVID-19 vaccine, which is urgently marketed, most others are in the early stages.^47^

**Fig. 3:**
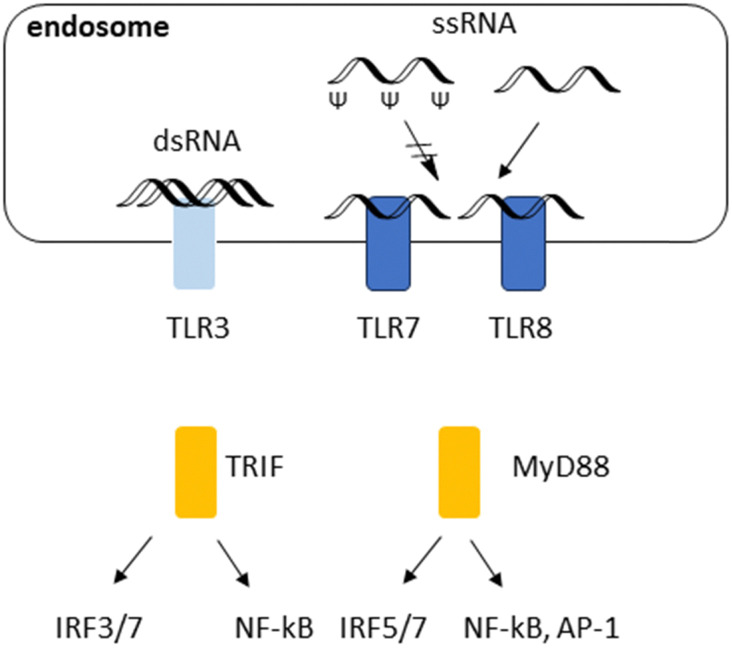
RNA response mechanisms and impact of Ψ modification. Endosomal RNA sensor TLR3 recognizes dsRNA and subsequently, activates the TRIF-dependent pathway to activate IRF3/7 and NF-κB. ssRNA binds to TLR7 and TLR8, activating MyD88, NF-kB, RF5/7 and AP-1. TLR, toll-like receptor; interferon regulatory factor, IRF; nuclear factor kappa-light-chain-enhancer of activated B cells; NF-kB, activation protein 1, AP-1.

Over the last decade, therapeutic mRNAs and mRNA vaccines have been encountering several major obstacles. First, long single (ss) or double stranded (ds) RNAs in the cytosol are commonly derived from the genome of RNA viruses or intermediate products that are generated during viral replication, leading to immune response.^48^ Introducing Ψ and *N*1-Me-Ψ has been a breakthrough to overcome this challenge. Immune responses to RNA and their inhibition with Ψ are illustrated in Fig. 3. TLR7 and TLR8 recognize single stranded (ss) RNA. These TLRs preferentially recognize polyuridine (polyU) and guanosine/uridine-rich (GU-rich) sequences. TLR7 and TLR8 also recognize RNA degradation products and require free guanosines and uridines, respectively, for maximum activation. Ψ and *N*1-methyl-Ψ could optimise mRNA performance by reduced immunogenicity and effective protein translation. Reduced binding to TLRs due to Ψ.

Delivery has been another main challenge to mRNA therapeutics and vaccines, that could not be solved solely with chemical modification of mRNA. mRNA is rapidly degraded *in vivo* and poorly uptaken by cells. Delivery systems like lipid nanoparticles (LNPs) have high efficiency, low toxicity and are applicable to various cell types. LNP-mRNA nanoparticle formulations have been successful and reached clinical applications.^44–46^ LNPs have encapsulation efficiency reaching 90%, and can be targeted through surface modification.^49,50^

On-going research on LNP mRNA covers multiple potential applications. Main direction in current trials is cancer vaccines. Very recently Moderna and Merck published successful results of phase IIb study on melanoma cancer vaccine.^51^ However it is a limiting factor that MC3 dLin-MC3-DMA (also known as C12-200) cationic formulation used in COVID-19 vaccine is patented and cannot be broadly used for other mRNA drug candidates. To overcome this, there are attempts to develop alternative formulations. Cationic lipid formulations using DOTAP (1,2-dioleoyl-3-trimethylammonium-propane), PEGylated lipids and DOPE (dioleoylphosphatidylethanolamine) are being tested.^52,53^

The delivery tools for mRNA are not limited to LNP. Naturally occurring vesicles, such as exosomes, can be used to carry mRNA.^52^ These vesicles are derived from cells and besides mRNA encapsulation, can facilitate cell-specific targeting. Encapsulation efficiency of exosomes is lower than for LNP, however exosomes are less toxic and have higher target specificity.^52^

Polymeric nanoparticles and dendrimers have been actively explored for the mRNA delivery.^53^ Polymeric nanoparticles, such as those made from polyethyleneimine (PEI), poly(lactic-*co*-glycolic acid) (PLGA), or chitosan, can form complexes with siRNA through electrostatic interactions. These nanoparticles protect mRNA from degradation and can enhance cellular uptake.^53,54^ Other examples of reported nanoparticles for mRNA include inorganic nanoparticles, such as gold or silica nanoparticles, hybrid nanoparticles, virus-like nanoparticles and peptide-based nanoparticles.^55–58^ They are extensively reviewed elsewhere.

Overall, there is no universal solution to all nucleic acid delivery tasks. Choosing the most suitable delivery system depends on the specific requirements of the therapeutic application, including the targeted tissue, desired release kinetics, and safety considerations.

### *N*1-Methylpseudouridine impact on COVID-19 mRNA vaccine

**Fig. 4:**
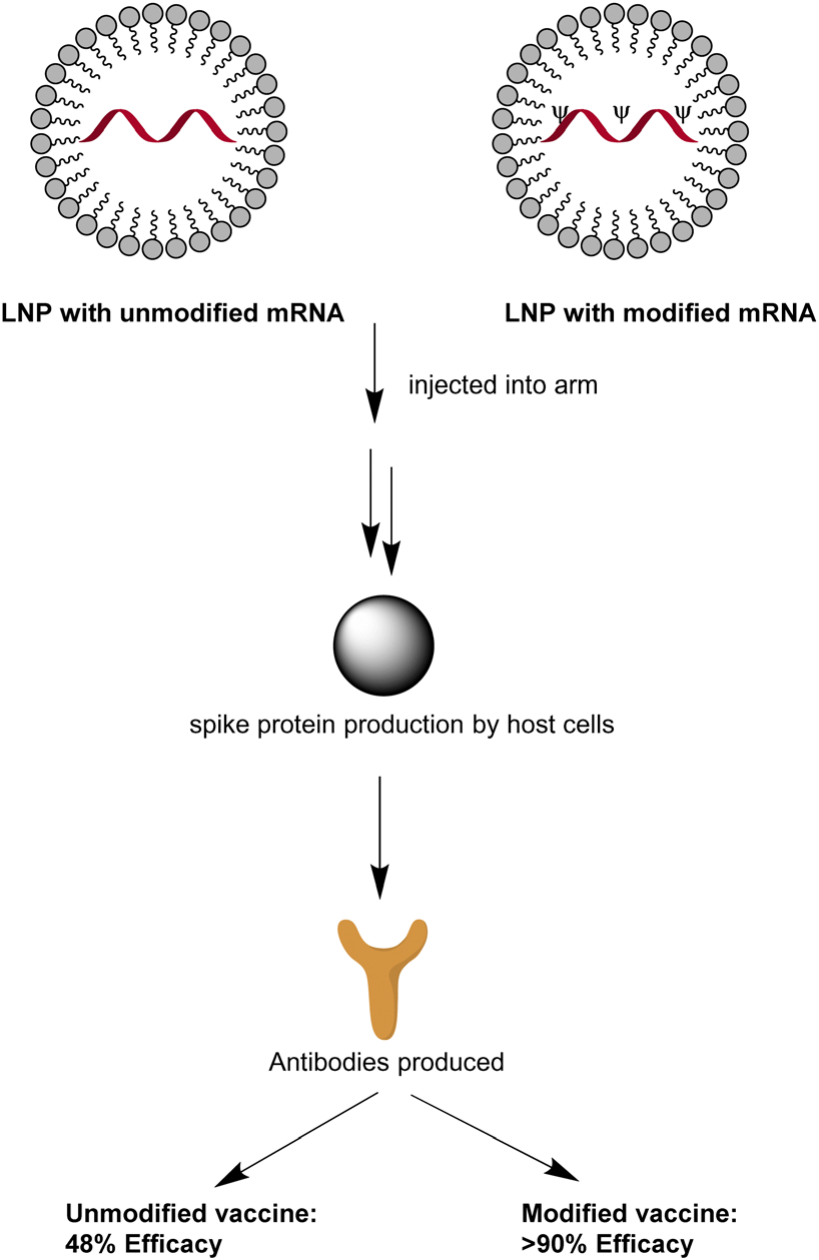
SARS-COVID-19 mRNA vaccination.

A key principle of the mRNA-based vaccination is that under low dosage, non-modified mRNA encodes the antigen while acting as an adjuvant (Fig. 4). Restricted to a maximum of 12 μg dosage by patients’ tolerance in late-stage clinical trials, unmodified mRNA vaccine CVnCoV maintained only 48% efficacy, regardless of the disease severity.^45^ In contrast, 30 μg Pfizer-BioNTech or 100 μg Moderna's mRNA vaccines could demonstrate around 95% high protection rate against COVID-19 after modification with *N*1-methyl-Ψ.^45^

Vaccines composed of unmodified and modified mRNA compared after being injected into the muscle of the upper arm.^45^ An immune response would be created and *N*1-methylpseudouridylated mRNA demonstrates over 90% efficacy against COVID-19 symptoms, much higher than the unmodified one which is beneath 50%.

Clearly, *N*1-methyl-Ψ has different impact on product protein translation compared to Ψ. According to Kim *et al.* (2022), *N*1-methyl-Ψ does not necessarily change the decoding accuracy in a reconstituted system.^59^ Neither would it increase the probability of miscoded peptides, nor stabilise the mismatched RNA-duplex formed. It only has a slight tendency in increasing errors when reverse transcription occurs.

### Remaining challenges in therapeutic mRNA development

mRNA development needs to be supported by research on mRNA structure since it is closely related to the function of mRNA. Nonetheless, mRNA structure prediction is still unreliable despite combining the usage of thermodynamic stability and evolutionary covariation information.^60^ The conserved mRNA structure could be predicted by the combination of three features: using significant covariation, negative evolutionary information and a plethora of probabilistic folding algorithms which incorporate those positive covariations into a single structure.^39^

**Fig. 5:**
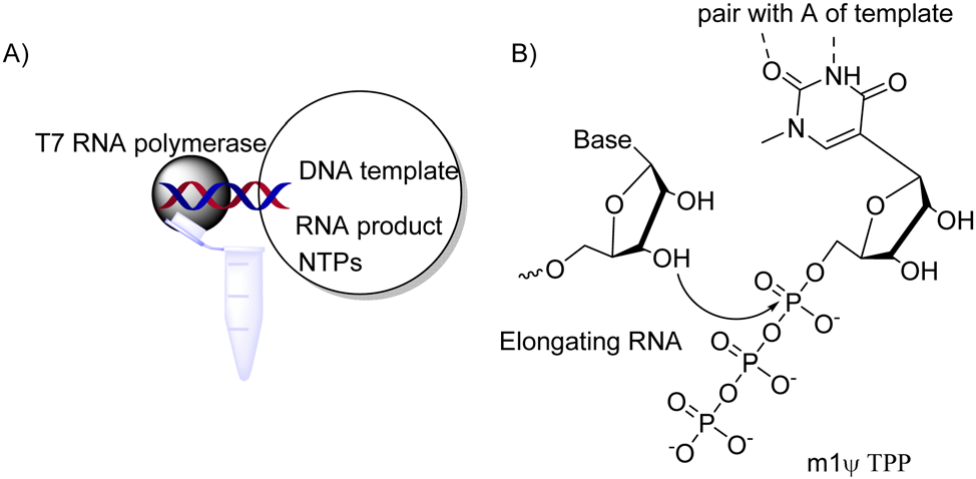
Producing *N*1-methyl-Ψ-modified mRNA by *in vitro* transcription. (A) Key components of enzymatic reaction and RNA product; NTP = nucleotide triphosphate;^63^ (B) enzymatic 3′ → 5′ elongation of RNA during *in vitro* transcription.

Another challenge is how to produce exogenous long mRNA while incorporating *N*1-methyl-Ψ flexibly on a large scale. Getting inspiration from nature and utilizing enzymes is an effective method. In this approach, fragments can become the building blocks of plasmids for COVID-19 vaccine synthesis. According to past studies, T7 polymerase could effectively build RNAs longer than 20 000 nucleotides perfectly without error^61,62^ while tolerating non-natural NTPs. m1Ψ triphosphate can be incorporated into RNA by polymerases providing long modified RNA molecules (Fig. 5).^63^ This allows producing large quantities of ling modified RNA, of tremendous benefit to RNA drug development and commercialization.

## Conclusions

Overall, mRNA therapy is a cutting-edge medical approach that uses synthetic mRNA molecules to prevent and treat various conditions. This technology has gained significant attention and recognition, particularly due to its role in the development of COVID-19 vaccines.^45^ mRNA therapy has the potential to treat a wide range of diseases, including infectious diseases, cancer, genetic disorders, and more.^64–66^ By introducing synthetic mRNA into the body, it's possible to instruct cells to produce specific proteins that can correct or combat disease. As to vaccine development, mRNA technology gained widespread attention during the COVID-19 pandemic when companies like Pfizer-BioNTech and Moderna developed highly effective COVID-19 vaccines using this approach.^45^ The vaccines contain mRNA that encodes the spike protein of the SARS-CoV-2 virus, which triggers an immune response, providing protection against the virus.^45^

One of the advantages of mRNA therapy is its rapid development and adaptability. Creating synthetic mRNA for a new target can be faster and more scalable than traditional vaccine or drug development processes. With LNP technology, efficient delivery can be achieved as well.^64–66^

Despite its promise, mRNA therapy faces challenges, including ensuring the stability and delivery of mRNA molecules to target cells, managing immune responses, and addressing potential side effects. These pitfalls can be overcome by using chemical analogues of RNA nucleotides. Pseudouridine (Ψ) is an important modification that is conserved naturally in the RNA structure. Studies showed that Ψ-modification allows mRNA to resist intrinsic immune responses^67^ and Ψ-derivatives could further improve mRNA properties, such as stability and efficacy of translation.

To apply this technology to vaccine and therapy development, deeper knowledge on the impact of Ψ and its analogues on RNA structure is required. Computational approaches, thermodynamics and structural investigations with *e.g.*, NMR would be significant next steps in this direction.

## Author contributions

The review is conceptualized by GHA Schiess and K. Astakhova. LLY Ho and K. Astakhova made the initial draft. All authors contributed to finalising the review.

## Conflicts of interest

There are no conflicts to declare.

## Supplementary Material
